# A fast tool for minimum hybridization networks

**DOI:** 10.1186/1471-2105-13-155

**Published:** 2012-07-02

**Authors:** Zhi-Zhong Chen, Lusheng Wang, Satoshi Yamanaka

**Affiliations:** 1 Division of Information System Design, Tokyo Denki University, Ishizaka, Hatoyama, Hiki, Saitama 359-0394, Japan; 2 Department of Computer Science, City University of Hong Kong, 83 Tat Chee Avenue, Kowloon, Hong Kong

## Abstract

**Background:**

Due to hybridization events in evolution, studying two different genes of a set of species may yield two related but different phylogenetic trees for the set of species. In this case, we want to combine the two phylogenetic trees into a hybridization network with the fewest hybridization events. This leads to three computational problems, namely, the problem of computing the minimum size of a hybridization network, the problem of constructing one minimum hybridization network, and the problem of enumerating a representative set of minimum hybridization networks. The previously best software tools for these problems (namely, Chen and Wang’s *HybridNet* and Albrecht *et al.*’s *Dendroscope 3*) run very slowly for large instances that cannot be reduced to relatively small instances. Indeed, when the minimum size of a hybridization network of two given trees is larger than 23 and the problem for the trees cannot be reduced to relatively smaller independent subproblems, then *HybridNet* almost always takes longer than 1 day and *Dendroscope 3* often fails to complete. Thus, a faster software tool for the problems is in need.

**Results:**

We develop a software tool in ANSI C, named *FastHN*, for the following problems: Computing the minimum size of a hybridization network, constructing one minimum hybridization network, and enumerating a representative set of minimum hybridization networks. We obtain *FastHN* by refining *HybridNet* with three ideas. The first idea is to preprocess the input trees so that the trees become smaller or the problem becomes to solve two or more relatively smaller independent subproblems. The second idea is to use a fast algorithm for computing the rSPR distance of two given phylognetic trees to cut more branches of the search tree in the exhaustive-search stage of the algorithm. The third idea is that during the exhaustive-search stage of the algorithm, we find two sibling leaves in one of the two forests (obtained from the given trees by cutting some edges) such that they are as far as possible in the other forest. As the result, *FastHN* always runs much faster than *HybridNet*. Unlike *Dendroscope 3*, *FastHN* is a single-threaded program. Despite this disadvantage, our experimental data shows that *FastHN* runs substantially faster than the multi-threaded *Dendroscope 3* on a PC with multiple cores. Indeed, *FastHN* can finish within 16 minutes (on average on a Windows-7 (x64) desktop PC with i7-2600 CPU) even if the minimum size of a hybridization network of two given trees is about 25, the trees each have 100 leaves, and the problem for the input trees cannot be reduced to two or more independent subproblems via cluster reductions. It is also worth mentioning that like *HybridNet*, *FastHN* does not use much memory (indeed, the amount of memory is at most quadratic in the input size). In contrast, *Dendroscope 3* uses a huge amount of memory. Executables of *FastHN* for Windows XP (x86), Windows 7 (x64), Linux, and Mac OS are available (see the Results and discussion section for details).

**Conclusions:**

For both biological datasets and simulated datasets, our experimental results show that *FastHN* runs substantially faster than *HybridNet* and *Dendroscope 3*. The superiority of *FastHN* in speed over the previous tools becomes more significant as the hybridization number becomes larger. In addition, *FastHN* uses much less memory than *Dendroscope 3* and uses the same amount of memory as *HybridNet*.

## Background

Constructing the evolutionary history of a set of species is an important problem in the study of biological evolution. Phylogenetic trees are used in biology to represent the ancestral history of a collection of existing species. This is appropriate for many groups of species. However, there are some groups for which the ancestral history cannot be represented by a tree. This is caused by processes such as hybridization, recombination, and lateral gene transfer. We refer to those processes as **reticulation** events. For this kind of groups of species, it is more appropriate to represent their ancestral history by rooted acyclic digraphs, where vertices of in-degree at least two represent reticulation events.

When studying the evolutionary history of a set of existing species, one can obtain a phylogenetic tree of the set of species with high confidence by looking at a segment of sequences or a set of genes. When looking at another segment of sequences, a different phylogenetic tree can be obtained with high confidence, too. This indicates that reticulation events may occur. Thus, we have the following problem: Given two rooted phylogenetic trees on a set of species that correctly represent the tree-like evolution of different parts of their genomes, what is the smallest number of reticulation events needed to explain the evolution of the species under consideration?

The subtree prune and regraft (rSPR) distance and the hybridization number are two important measures for evolutionary tree comparison and hybridization network construction. Since both problems are NP-hard [1-3], it is challenging to develop programs that can give exact solutions when the two given trees are large or have a large rSPR distance or hybridization number. Previously, several software packages have been developed for these problems [4-9]. This new breakthrough brings us a hope that one can routinely solve these hard problems for two given large trees. However, the previously fastest software packages can still take hours to finish even when the given trees are of moderate sizes. Thus, a faster software tool for these problems is in need.

In general, there may exist two or more minimum hybridization networks displaying two given phylogenetic trees *T*_1_ and *T*_2_ with the same leaf set *X*. In some cases, we may want to enumerate all minimum hybridization networks displaying both *T*_1_ and *T*_2_. Unfortunately, it is not hard to construct two example phylogenetic trees *T*_1_ and *T*_2_ such that there are too many minimum hybridization networks displaying both *T*_1_ and *T*_2_. So, we instead want to enumerate only a *representative set* of minimum hybridization networks displaying both *T*_1_ and *T*_2_. Here, a hybridization network *Nrepresents* another hybridization network *N’* if for every pair (*x,y*) of species in *Xx* and *y* fall into the same connected component of *F*^*N*^ if and only if they fall into the same connected component of *F’*_*N*_, where *F*^*N*^ (respectively, *F’*_*N*_) is the forest obtained from *N* (respectively, *N’*) by removing all the edges entering reticulate nodes. *HybridNet*[6] and *Dendroscope 3*[4] are able to enumerate a representative set of of minimum hybridization networks for two given phylogenetic trees. If the problem for the two given trees can be reduced to relatively smaller independent subproblems (by so-called “cluster reductions”), *Dendroscope* is much faster than *HybridNet*; otherwise, the two have almost the same speed. Unfortunately, both tools run very slowly when the minimum hybridization number of a hybridization network of two given trees is large (say, larger than 23) and the problem for the trees cannot be reduced to relatively smaller independent subproblems. Thus, a much faster tool is in need.

## Results and discussion

We have developed a new tool (called *FastHN*) for the problem of enumerating a representative set of minimum hybridization networks of two given phylognetic trees. Of course, *FastHN* can also compute the minimum hybridization number of a hybridization network of two given phylognetic trees and construct a single minimum hybridization network of two given phylognetic trees. *FastHN* is implemented in ANSI C and is available at http://rnc.r.dendai.ac.jp/∼chen/fastHN.html, or http://www.cs.cityu.edu.hk/∼lwang/software/FastHN/fastHN.html, where one can download executables for Windows XP (x86), Windows 7 (x64), Linux, and Mac OS.

After downloading *FastHN*, one can run it as follows:

FastHN T1 T2 OPTION HEURISTIC or simply FastHN T1 T2 OPTION

Here, T1 and T2 are two text files each containing a phylogenetic tree in the Newick format (ended with a semicolon). The label of each leaf in an input tree should be a string consisting of letters in {0,1,…,9,*a*,*b*,…,*z*,*A*,*B*,…,*Z*,_,.}. There is no limit on the length of the label of each leaf.

OPTION is a string in the set {HN, MAAF, MAAFs} controlling the output as follows: 

· HN: The output is the hybridization number of T1 and T2.

· MAAF: The output is one MAAF of T1 and T2 together with one minimum hybridization network for the MAAF.

· MAAFs: The output is all MAAFs of T1 and T2 together with one minimum hybridization network for each MAAF.

*FastHN* outputs an MAAF (respectively, MAF) by printing out the leaf sets of the trees in the MAAF (respectively, MAF), while it outputs a hybridization network in its extended Newick format [10]. When OPTION is MAAFs (respectively, MAFs), *FastHN* outputs the MAAFs (respectively, MAFs) without repetition. We remind the reader that one can view a tree in the Newick format and a network in the extended Newick format by using Dendroscope due to [11].

HEURISTIC is a 3-bit binary string specifying the version of *FastHN* as follows: 

· The first bit is 1 if and only if *FastHN* adopts initial cluster reductions.

· The second bit is 1 if and only if *FastHN* adopts Heuristic 1.

· The last bit is 1 if and only if *FastHN* adopts Heuristic 2.

HEURISTIC can be omitted; in that case, it is set to be 111.

To compare the efficiency of *FastHN* with the previous bests (namely, *HybridNet*[6] and *Dendroscope 3*[4]), we have run them on both simulated datasets and biological datasets for the problem of computing all MAAFs of two given phylogenetic trees. The experiment has been performed on a Windows-7 (x64) desktop PC with i7-2600 CPU and 4GB RAM. It is worth mentioning that in our experiments, we have used the *total elapsed time* (rather than the *CPU time*) to measure the running time of *FastHN*. Since *FastHN* is single-threaded, its total elapsed time is usually more than its CPU time. In contrast, *Dendroscope 3* is multi-threaded, its total elapsed time can be less than its CPU time. Because it is not clear how *Dendroscope 3* measures its running time, we have *pessimistically* measured the running time of *FastHN* using the total elapsed time (in order to do a fair comparison with *Dendroscope 3*).

### Simulated data

To generate simulated datasets, we use a program due to Beiko and Hamilton [12]. To obtain a pair (*T, T’*) of trees, their program first generates *T* randomly and then obtains *T’* from *T* by performing a specified number *r* (say, 20) of random rSPR operations on *T*. Recall that an *rSPR operation* on a tree *T* first removes an edge (*p,c*) from *T*, then contracts *p* (the vertex of out-degree 1 resulting from the removal of edge (*p,c*)), and further re-attaches the subtree rooted at *c* to an edge (*p’,c’*) of *T* (by introducing a new vertex *m’*, splitting edge (*p’,c’*) into two edges (*p’,m’*) and (*m’,c’*), and adding a new edge (*m’,c*)). So, the actual rSPR distance of *T* and *T’* is at most *r*. Moreover, the hybridization number of *T* and *T’* can be *r*, smaller than *r*, or larger than *r*.

**Figure 1:**
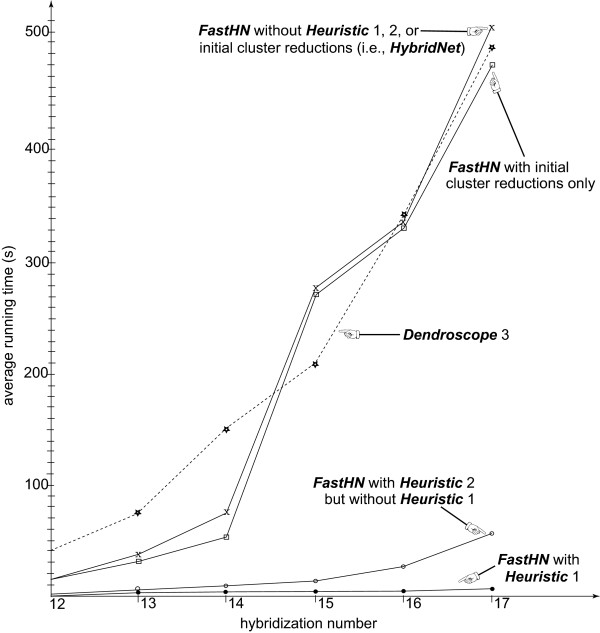
***FastHN*vs. *Dendroscope 3* on simulated data.**

We first use Beiko and Hamilton’s program to generate 120 pairs of trees each of which has **100** leaves. The first (respectively, second) 60 pairs are generated by setting r = 14 (respectively, r = 17). It turns out that among the 120 generated tree-pairs, 6 (respectively, 22, 33, 11, 21, or 27) tree-pairs have hybridization number 12 (respectively, 13, 14, 15, 16, or 17). Figure 1 summarizes the average running time of the programs for the generated tree-pairs, where each average is taken over those tree-pairs with the same hybridization number. As can be seen from the figure, *FastHN* with Heuristic 1 and/or Heuristic 2 is much faster than *HybridNet* and *Dendroscope 3*. This difference in speed becomes more significant as the hybridization number becomes larger. Moreover, Heuristic 1 contributes the most to the saving of running time. Indeed, when Heuristic 1 is used, both Heuristic 2 and initial cluster reductions do not help much. It is worth noting that Beiko and Hamilton’s program tends to create a pair of trees without a relatively large common clusters. This is why initial cluster reductions do not help much for tree-pairs randomly generated by their program.

The comparison is done on 120 randomly generated tree-pairs with relatively small hybridization numbers, where each tree has 100 leaves. If the running time of a program for a tree-pair is more than 600 seconds, then it has been rounded down to 600 seconds. Among the 120 pairs, *Dendroscope 3* takes more than 600 seconds for 28 pairs, *FastHN* without Heuristic 1, 2, or initial cluster reductions takes more than 600 seconds for 20 pairs, *FastHN* with only initial cluster reductions takes more than 600 seconds for 18 pairs, and *FastHN* with Heuristic 1 or 2 takes less than 60 seconds for every pair.

**Figure 2:**
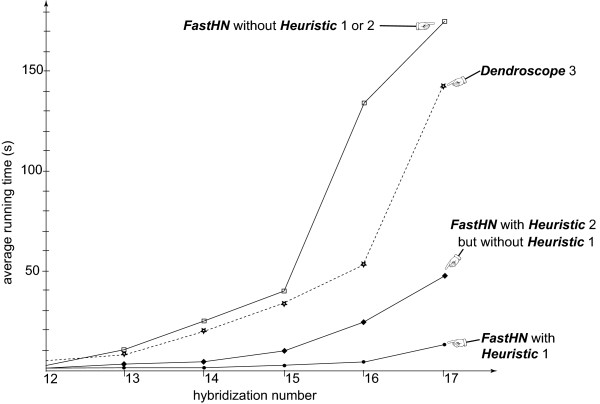
***FastHN*vs.** Dendroscope 3 on simulated data.

To test how the number of leaves in an input tree influences the running time of the algorithms, we next use Beiko and Hamilton’s program to generate 120 pairs of trees each of which has **50** leaves. The first (respectively, second) 60 pairs are generated by setting r = 14 (respectively, r = 17). It turns out that among the 120 generated tree-pairs, 3 (respectively, 17, 26, 26, 20, 20, or 6) tree-pairs have hybridization number 11 (respectively, 12, 13, 14, 15, 16, or 17). Moreover, for each *h*∈{9,10}, there is exactly one generated tree-pair with hybridization number *h*. Figure 2 summarizes the average running time of the programs for those generated tree-pairs with hybridization number in the range [12 .. 17], where each average is taken over those tree-pairs with the same hybridization number. As can be seen from the figure, the superiority of *FastHN* over *HybridNet* and *Dendroscope 3* remains the same (as in Figure 1) if Heuristic 1 or 2 is used. Moreover, Heuristic 1 contributes the most to the saving of running time.

The comparison is done on 120 randomly generated tree-pairs with relatively small hybridization numbers, where each tree has 50 leaves.

**Figure 3:**
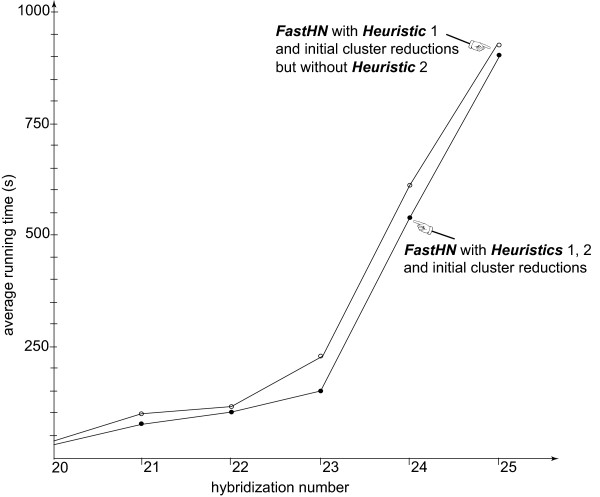
**Average running time of*FastHN*on simulated data.**

To compare the performance of the algorithms for tree-pairs with relatively large hybridization numbers, we further use Beiko and Hamilton’s program to generate 60 pairs of trees by setting r = 25, where each tree has **100** leaves. It turns out that among the 60 generated tree-pairs, 4 (respectively, 10, 15, 18, or 12) tree-pairs have hybridization number 21 (respectively, 22, 23, 24, or 25). Moreover, there is exactly one generated tree-pair with hybridization number 20. Figure 3 summarizes the average running time of the two best versions of *FastHN* for the generated tree-pairs, where each average is taken over those tree-pairs with the same hybridization number. As can be seen from the figure, both versions take less than 16 minutes (on average) even when the hybridization number is as large as 25, while *FastHN* with both Heuristics 1 and 2 is the faster version. In contrast, *Dendroscope 3* fails to complete for each of the 60 tree-pairs.

The time is measured on 60 randomly generated tree-pairs with relatively large hybridization numbers, where each tree has 100 leaves. (**Note:***Dendroscope 3* fails to complete for each of the 60 pairs.)

### Biological data

We use the Poaceae dataset from the Grass Phylogeny Working Group [13]). The dataset contains sequences for six loci: internal transcribed spacer of ribosomal DNA (ITS); NADH dehydrogenase, subunit F (ndhF); phytochrome B (phyB); ribulose 1,5-biphosphate carboxylase/oxygenase, large subunit (rbcL); RNA polymerase II, subunit ^*β*′^ (rpoC2); and granule bound starch synthase I (waxy). The Poaceae dataset was previously analyzed by [14], who generated the inferred rooted binary trees for these loci. See Table 1 for the experimental results. In this table, column *pair* shows the tree-pairs, column *#taxa*shows the number of leaves in an input tree, and column *h* shows the hybridization number of each tree-pair. Moreover, columns *FastHN* and *Dendroscope* show the running times (in seconds) of *FastHN* and *Dendroscope 3*, respectively. Furthermore, column *FastHN* has 8 sub-columns each labeled by 3 bits, where the first (respectively, middle, or the last) bit is 1 if and only if initial cluster reductions (respectively, Heuristic 1, or Heuristic 2) are adopted. In particular, the sub-column labeled 000 corresponds to *HybridNet*.

**Table 1:** *FastHN* vs. *Dendroscope 3* on 15 tree-pairs in the Poaceae dataset

			***FastHN***								
***pair***	***#taxa***	***h***	**000**	**001**	**010**	**011**	**100**	**101**	**110**	**111**	***Dendroscope***
ndhF,phyB	40	14	19	15	7	5	1	1	2	1	2
ndhF,rbcL	36	13	5	5	6	5	1	1	1	1	1
ndhF,rpoC2	34	12	2	2	1	1	1	1	1	1	1
ndhF,waxy	19	9	1	1	1	1	1	1	1	1	1
ndhF,ITS	46	19	556	537	53	97	1	1	1	1	2
phyB,rbcL	21	4	1	1	1	1	1	1	1	1	1
phyB,rpoC2	21	7	1	1	1	1	1	1	1	1	1
phyB,waxy	14	3	1	1	1	1	1	1	1	1	1
phyB,ITS	30	8	1	1	1	1	1	1	1	1	1
rbcL,rpoC2	26	13	2	2	1	1	1	1	1	1	1
rbcL,waxy	12	7	1	1	1	1	1	1	1	1	1
rbcL,ITS	29	14	10	9	2	2	4	3	1	1	9
rpoC2,waxy	10	1	1	1	1	1	1	1	1	1	1
rpoC2,ITS	31	15	14	13	14	8	2	2	1	1	2
waxy,ITS	15	8	1	1	1	1	1	1	1	1	1

As can be seen from Table 1, for most of the tree-pairs, there is not much difference in speed between *Dendroscope 3* and *FastHN* with initial cluster reductions. This is because most of the tree-pairs have small hybridization numbers. For the tree-pair (ndhf, ITS), *FastHN* with cluster reductions runs substantially faster than *FastHN* without cluster reductions. This is because the problem for this pair can be reduced to two tree-pairs of roughly equal sizes by initial cluster reductions in the preprocessing stage of the algorithm.

### Discussion

Roughly speaking, *FastHN* consists of two stages, namely, the preprocessing stage and the exhaustive-search stage. In the preprocessing stage, *FastHN* performs only subtree reductions and cluster reductions. Indeed, other kinds of reductions are also known. One of them is chain reduction [15]. Performing chain reductions on the input trees results in trees whose nodes are weighted. Unfortunately, it seems that Whidden *et al.*’s *O*(2.4^2*d*^*n*)-time algorithm for computing the rSPR distance *d* of two given phylognetic trees with *n* leaves does not work when the trees are weighted. This is why *FastHN* does not perform chain reductions.

In the exhaustive-search stage, *FastHN* also performs subtree reductions whenever possible, but does not perform cluster reductions. The main reason of not performing cluster reductions in the 2nd stage is that performing a cluster reduction is too time-consuming (namely, takes *O*(^*n*2^) time, where *n* is the number of leaves in the trees).

When running *FastHN*, one can decide whether to adopt initial cluster reductions, Heuristic 1, or Heuristic 2. If two input trees have relatively large common clusters, performing initial cluster reductions on them lead to solving independent and significantly smaller subproblems. So, we should always choose to adopt initial cluster reductions. Moreover, as can be seen from our simulated results, we should always choose to adopt Heuristic 1 because it enables the algorithm to save a lot of time by cutting more branches of the search tree in the exhaustive-search stage. Our simulated results also show that adopting both Heuristics 1 and 2 makes *FastHN* run faster (on average) than adopting only Heuristic 1. Thus, in general, we should choose to adopt Heuristic 2 as well. However, in our experiments, we have found some tree-pairs for which *FastHN* with Heuristic 1 but without Heuristic 2 runs significantly faster than *FastHN* with both Heuristics 1 and 2. Hence, as long as Heuristic 1 is adopted, there is still room to decide whether to adopt Heuristic 2 as well.

## Conclusions

Our experiments show that *FastHN* runs substantially faster than the previously best tools (namely, *HybridNet* and *Dendroscope 3*). The fast speed of *FastHN* originates from two key new ideas (which have not been used to solve the problems before, as far as we know): 

· We use a fast algorithm for computing the rSPR distance of two given phylognetic trees to cut more branches of the search tree during the exhaustive-search stage of *FastHN*.

· During the exhaustive-search stage of *FastHN*, we always try to find a pair of sibling leaves in one of the two forests (obtained from the given trees by cutting some edges) such that the two leaves is as far apart as possible in the other forest.

## Methods

Throughout this section, a *rooted forest* always means a directed acyclic graph in which every node has in-degree at most 1 and out-degree at most 2.

Let *F* be a rooted forest. The *roots* (respectively, *leaves*) of *F* are those nodes whose in-degrees (respectively, out-degrees) are 0. The *size* of *F*, denoted by —*F*—, is the number of roots in *F* minus 1. A node *v* of *F* is *unifurcate* if it has only one child in *F*. If a root *v* of *F* is unifurcate, then *contracting v in F* is the operation that modifies *F* by deleting *v*. If a non-root node *v* of *F* is unifurcate, then *contracting v in F* is the operation that modifies *F* by first adding an edge from the parent of *v* to the child of *v* and then deleting *v*.

For convenience, we view each node *u* of *F* as an ancestor and descendant of *u* itself. A node *u* is *lower than* another node *v*≠*u* in *F* if *u* is a descendant of *v* in *F*. The *lowest common ancestor* (LCA) of a set *U* of nodes in *F* is the lowest node *v* in *F* such that for every node *u*∈*U*, *v* is an ancestor of *u* in *F*. For a node *v* of *F*, the *subtree of F rooted at v* is the subgraph of *F* whose nodes are the descendants of *v* in *F* and whose edges are those edges connecting two descendants of *v* in *F*. If *v* is a root of *F*, then the subtree of *F* rooted at *v* is a *component tree* of *F*. *F* is a *rooted tree* if it has only one root.

A *rooted binary forest* is a rooted forest in which the out-degree of every non-leaf node is 2. Let *F* be a rooted binary forest. *F* is a *rooted binary tree* if it has only one root. If *v* is a non-root node of *F* with parent *p* and sibling *u*, then *detaching the subtree of F rooted at v* is the operation that modifies *F* by first deleting the edge (*p,v*) and then contracting *p*. A *detaching operation* on *F* is the operation of detaching the subtree of *F* rooted at a non-root node.

### Hybridization networks and phylogenetic trees

Let *X* be a set of existing species. A *hybridization network* on *X* is a directed acyclic graph *N* in which the set of nodes of out-degree 0 (still called the *leaves*) is *X*, each non-leaf node has out-degree 2, there is exactly one node of in-degree 0 (called the *root*), and each non-root node has in-degree larger than 0. Note that the in-degree of a non-root node in *N* may be larger than 1. A node of in-degree larger than 1 in *N* is called a *reticulation node* of *N*. Intuitively speaking, a reticulation node corresponds to a reticulation event. The *hybridization number* of a reticulation node in *N* is its in-degree in *N* minus one. The *hybridization number* of *N* is the total hybridization number of reticulation nodes in *N*.

A *phylogenetic tree* on *X* is a rooted binary tree whose leaf set is *X*. A hybridization network *N* on *Xdisplays* a phylogenetic tree *T* on *X* if *N* has a subgraph *M* such that *M* is a rooted tree, the root of *M* has exactly two children in *M*, and modifying *M* by contracting its unifurcate nodes yields *T*. A *hybridization network* of two phylogenetic trees *T*_1_ and *T*_2_ on *X* is a hybridization network *N* on *X* such that *N* displays both *T*_1_ and *T*_2_. A hybridization network of *T*_1_ and *T*_2_ is *minimum* if its hybridization number is minimized among all hybridization networks of *T*_1_ and *T*_2_. Obviously, if *N* is a minimum hybridization network of *T*_1_ and *T*_2_, then the in-degree of every reticulation node in *N* is exactly 2 and hence the hybridization number of *N* is equal to the number of reticulation nodes in *N*. For convenience, we define the *hybridization number* of *T*_1_ and *T*_2_ to be the minimum hybridization number of a hybridization network of *T*_1_ and *T*_2_.

We are now ready to define one problem studied in this paper: 

Hybridization Network Construction (HNC):

· **Input:** Two phylogenetic trees *T*_1_ and *T*_2_ on the same set *X* of species.

· **Goal:** To construct a minimum hybridization network of *T*_1_ and *T*_2_.

### Agreement forests

Throughout this subsection, let *T*_1_ and *T*_2_ be two phylogenetic trees on the same set *X* of species. If we can apply a sequence of detaching operations on each of *T*_1_ and *T*_2_ so that they become the same forest *F*, then we refer to *F* as an *agreement forest* (AF) of *T*_1_ and *T*_2_. A *maximum agreement forest* (MAF) of *T*_1_ and *T*_2_ is an agreement forest of *T*_1_ and *T*_2_ whose size is minimized over all agreement forests of *T*_1_ and *T*_2_. The size of an MAF of *T*_1_ and *T*_2_ is called the *rSPR distance* between *T*_1_ and *T*_2_. The following lemma is shown in [16].

#### Lemma 1

[16] Given two phylogenetic trees *T*_1_ and *T*_2_, we can compute the rSPR distance between *T*_1_ and *T*_2_ in *O*(2.4^2*d*^*n*) time, where *n* is the number of leaves in *T*_1_ and *T*_2_ and *d* is the rSPR distance between *T*_1_ and *T*_2_.

Let *F* be an agreement forest of *T*_1_ and *T*_2_. Obviously, for each *i*∈{1,2}, the leaves of *T*_*i*_ one-to-one correspond to the leaves of *F*. For convenience, we hereafter identify each leaf *v* of *F* with the leaf of *T*_*i*_ corresponding to *v*. Similarly, for each *i*∈{1,2}, the non-leaf nodes of **F** correspond to distinct non-leaf nodes of *T*_*i*_. More precisely, a non-leaf node *u* of *F* corresponds to the LCA of {_*v*1_,…,_*vℓ*_} in *T*_*i*_, where *v*_1_, …, _*vℓ*_are the leaf descendants of *u* in *F*. Again for convenience, we hereafter identify each non-leaf node *u* of *F* with the non-leaf node of *T*_*i*_ corresponding to *u*. With these correspondences, we can use *F*, *T*_1_, and *T*_2_ to construct a directed graph *G*_*F*_ as follows: 

· The nodes of *G*_*F*_ are the roots of *F*.

· For every two roots _*r*1_and _*r*2_of *F*, there is an edge from _*r*1_to _*r*2_in *G*_*F*_ if and only if _*r*1_is an ancestor of _*r*2_in *T*_1_ or *T*_2_.

We refer to *G*_*F*_ as the *decision graph associated withF*. If *G*_*F*_ is acyclic, then *F* is an *acyclic agreement forest* (AAF) of *T*_1_ and *T*_2_; otherwise, *F* is a *cyclic agreement forest* (CAF) of *T*_1_ and *T*_2_. If *F* is an AAF of *T*_1_ and *T*_2_ and its size is minimized over all AAFs of *T*_1_ and *T*_2_, then *F* is a *maximum acyclic agreement forest* (MAAF) of *T*_1_ and *T*_2_. Note that our definition of an AAF is the same as those in [15,17] but is different from that in [16]. Moreover, it is known that the size of an MAAF of *T*_1_ and *T*_2_ is equal to the hybridization number of *T*_1_ and *T*_2_[18]. The following lemma is shown in [19]:

#### Lemma 2

[19] Suppose that *C* is a cycle of *G*_*F*_ and *r*_1_, …, _*rℓ*_are the nodes of *C*. Then, each _*rj*_∈{_*r*1_,…,_*rℓ*_} has two children *u*_*j*_ and *u’*_*j*_ in *F*. Moreover, for every non-root node *v* of *F* not contained in {_*u*1_$u_{1}^{\prime }$,…,_*uℓ*_$u_{\ell }^{\prime }$}, *C* remains a cycle in *G*_*F*_ after *F* is modified by detaching the subtree of *F* rooted at *v*.

Let *N* be a minimum hybridization network of *T*_1_ and *T*_2_. Suppose that we modify *N* to obtain a forest *F*(*N*) by first removing all edges entering reticulation nodes, then removing those nodes *v* such that neither *v* nor its descendants are in *X*, and further contracting all unifurcate nodes. Obviously, *F*(*N*) is an AAF of *T*_1_ and *T*_2_ and the size of *F*(*N*) is exactly the hybridization number of *N*. So, each MAAF *F’* of *T*_1_ and *T*_2_*represents* the set of all minimum hybridization networks *N* such that *F*(*N*) is the same as *F’*. Thus, to enumerate a representative set of minimum hybridization networks of *T*_1_ and *T*_2_, the idea in previous work [6] has been to enumerate all MAAFs of *T*_1_ and *T*_2_ and construct a minimum hybridization network for each enumerated MAAF. Since we can easily use an MAAF of *T*_1_ and *T*_2_ to construct a hybridization network displaying *T*_1_ and *T*_2_[6], the difficulty is in how to enumerate all MAAFs of *T*_1_ and *T*_2_.

We are now ready to define another problem studied in this paper: 

Hybridization Network Enumeration (HNE):

· **Input:** Two phylogenetic trees *T*_1_ and *T*_2_ on the same set *X* of species.

· **Input:** Two phylogenetic trees *T*_1_ and *T*_2_ on the same set *X* of species.

· **Goal:** To enumerate all MAAFs of *T*_1_ and *T*_2_ and construct a minimum hybridization network of *T*_1_ and *T*_2_ from each MAAF of *T*_1_ and *T*_2_.

Basically, HNE is the problem of enumerating a representative set of minimum hybridization networks of two given phylogenetic trees. As in previous studies [5,6,8], when we consider HNC and HNE, we always assume that each given phylogenetic tree has been modified by first introducing a new root and a dummy leaf and then letting the old root and the dummy leaf be the children of the new root.

The following lemma is shown in [19]:

#### Lemma 3

[19] The dummy leaf alone does not form a component tree of an MAAF of *T*_1_ and *T*_2_.

### Extending Whidden *et al.*’s Algorithm

Throughout this subsection, let *T*_1_ and *T*_2_ be two phylogenetic trees on the same set *X* of species. We sketch the fastest known algorithm (due to Whidden *et al.*[16]) for computing an MAF of *T*_1_ and *T*_2_, and then state a slight extension of the algorithm that will be used in our algoirthm for HNE.

The basic idea behind Whidden *et al.*’s algorithm is as follows. For k = 0, 1, 2, …(in this order), we try to find an AF of *T*_1_ and *T*_2_ of size *k* and stop immediately once such an AF is found. To find an AF of *T*_1_ and *T*_2_ of size *k*, we start by setting _*F*1_=_*T*1_ and _*F*2_=_*T*2_ and associating a *label set*{*x*} to each leaf *x* of *F*_1_ and *F*_2_. We then repeatedly modify *F*_1_ and *F*_2_ (until either |_*F*1_|>*k* or *F*_1_ becomes a forest without edges) as follows. We find two arbitrary sibling leaves *u* and *v* in *F*_2_. If *u* and *v* are also siblings in *F*_1_, then we modify *F*_1_ and *F*_2_ separately by merging the identical subtrees of *F*_1_ and *F*_2_ rooted at the parent of *u* and *v* each into a single leaf whose label set is the union of the label sets of *u* and *v*. On the other hand, if *u* and *v* are not siblings in *F*_1_, then we distinguish three cases as follows. *Case 1:u* and *v* are in different component trees of *F*_1_. In this case, in order to transform *F*_1_ and *F*_2_ into an AF of *T*_1_ and *T*_2_, we have two choices to modify them, namely, by either detaching the subtree rooted at *u* or detaching the subtree rooted at *v*. *Case 2:u* and *v* are in the same component tree of *F*_1_ and either (1) *u* and the parent of *v* are siblings in *F*_1_ or (2) *v* and the parent of *u* are siblings in *F*_1_. In this case, if (1) (respectively, (2)) holds, then we modify *F*_1_ by detaching the subtree rooted at the sibling of *v* (respectively, *u*). *Case 3:u* and *v* are in the same component tree of *F*_1_ and neither (1) nor (2) in Case 2 holds. In this case, in order to transform *F*_1_ and *F*_2_ into an AF of *T*_1_ and *T*_2_, we have three choices to modify them. The first two choices are the same as those in Case 1. In the third choice, we modify *F*_1_ by detaching the subtrees rooted at those non-root nodes *w* such that the parent of *w* appears on the (not necessarily directed) path between *u* and *v* in *F*_1_ but *w* does not.

By the above three cases, we always have the following: 

· |_*F*1_|≥|_*F*2_|.

· All component trees of *F*_2_ except at most one have no edges.

· For each component tree _*Γ*2_of *F*_2_ without edges, *F*_1_ has a component tree _*Γ*1_without edges such that the label sets associated with the unique leaves of _*Γ*1_and _*Γ*2_are identical.

Once |_*F*1_| becomes larger than *k*, we know that *F*_1_ and *F*_2_ have no AF of size *k*. On the other hand, once *F*_1_ becomes a forest without edges, we can use the label sets *L*(*v*) of the leaves *v* of *F*_1_ to obtain an AF of *T*_1_ and *T*_2_ of size |_*F*1_| by modifying *T*_1_ as follows. For each leaf *v* of *F*_1_ such that *L*(*v*) does not contain the dummy leaf, detach the subtree of *T*_1_ rooted at the LCA of the leaves in *L*(*v*).

Now, we are now ready to make a key observation in this paper. By (b) and (c) in the above, Whidden *et al.*’s MAF algorithm can actually be used to solve the following slightly more general problem in *O*(2.4^2*k*^*n*) time: 

rSPR Distance Checking (rSPRDC):

· **Input:**(_*T*1_,_*T*2_,*k*,_*F*1_,_*F*2_), where *T*_1_ and *T*_2_ are two phylogenetic trees on the same set *X* of species, *k* is an integer, *F*_1_ (respectively, *F*_2_) is a rooted forest obtained from *T*_1_ (respectively, *T*_2_) by performing zero or more detaching operations, and every component tree of *F*_2_ except at most one is identical to a component tree of *F*_1_.

· **Goal:** To decide if performing *k* more detaching operations on *F*_1_ leads to an AF of *T*_1_ and *T*_2_.

Finally, if we want to enumerate all MAFs of *T*_1_ and *T*_2_, then we need to modify Whidden *et al.*’s algorithm as follows. First, we do not distinguish Cases 2 and 3 because modifying *F*_1_ as in Case 2 may lose some MAF of *T*_1_ and *T*_2_. Moreover, whenever an AF of *T*_1_ and *T*_2_ of size *k* is found, we do not stop immediately and instead continue to find other AFs of *T*_1_ and *T*_2_ of size *k*. The resulting algorithm runs more slowly, namely, in *O*(^3*k*^*n*) time.

### Speeding up *HybridNet*

Throughout this subsection, let *T*_1_ and *T*_2_ be two phylogenetic trees on the same set *X* of species. We first sketch how *HybridNet* enumerates all MAAFs of *T*_1_ and *T*_2_, and then explain how to speed it up.

First, we need several definitions. For a rooted forest *F*, we use (*F*) to denote the family of the leaf sets of the component trees of *F*. Let *F* and *F’* be two forests each obtained by performing zero or more detaching operations on *T*_1_. If *F*≠^*F″*^and for every set Y∈ℒ(F), there is a set $Y^{\prime \prime }\in ℒ(F^{\prime \prime })$ with $Y\subseteq Y^{\prime \prime }$, then we say that *F* is *finer thanF’* and *F’* is *coarser thanF*.

To enumerate all MAAFs of *T*_1_ and *T*_2_, the idea behind *HybridNet* is to design an algorithm for the following problem: 

Generalized Agreement Forest (GAF)

· **Input:**(_*T*1_,_*T*2_,*k*,_*F*1_), where *T*_1_ and *T*_2_ are two phylogenetic trees on the same set *X* of species, *k* is an integer, and *F*_1_ is a rooted forest obtained from *T*_1_ by performing zero or more detaching operations.

· **Goal:** To find a sequence of AFs of *T*_1_ and *T*_2_ including all AFs *F* of *T*_1_ and *T*_2_ such that (1) *F* can be obtained by performing at most *k* detaching operations on *F*_1_ (or equivalently, at most |_*F*1_| + *k*detaching operations on *T*_2_) and (2) no AF of *T*_1_ and *T*_2_ is finer than *F*_1_ and coarser than *F*.

In the supplementary material of [19], an *O*(^3*k*^*n*)-time algorithm for solving GAF is detailed. The algorithm differs from Whidden *et al.*’s algorithm for enumerating all MAFs of *T*_1_ and *T*_2_ only in that we start with *F*_1_ (as it is given) and _*F*2_=_*T*2_(instead of starting with _*F*1_=_*T*1_and _*F*2_=_*T*2_) and then repeatedly modify *F*_1_ and *F*_2_ until either |_*F*1_|>*k* + _*k*0_or *F*_1_ becomes a forest without edges, where *k*_0_ is the original size of *F*_1_. Now, we are now ready to make two other key observations in this paper. To speed up Chen and Wang’s algorithm for solving GAF, we modify it as follows: 

· **Heuristic 1:** Every time before we start to make multiple choices of modifying *F*_1_ and *F*_2_, we call the algorithm for rSPRDC in Lemma 1 on input (_*T*1_,_*T*2_,*k*−|_*F*1_| + _*k*0_,_*F*1_,_*F*2_) to check if performing *k*−|_*F*1_| + _*k*0_more detaching operations on *F*_1_ leads to an AF of *T*_1_ and *T*_2_.

As the result, if we know that performing *k*−|_*F*1_| + _*k*0_more detaching operations on *F*_1_ does not lead to an AF of *T*_1_ and *T*_2_, then no more choice of modifying *F*_1_ and *F*_2_ is necessary; otherwise, we proceed to make multiple choices of modifying *F*_1_ and *F*_2_ the same as before but with the following difference: 

· **Heuristic 2:** Instead of selecting two arbitrary sibling leaves *u* and *v* in *F*_2_ (cf. the Extending Whidden *et al.*’s Algorithm section), we select two sibling leaves *u* and *v* in *F*_2_ such that they are as far apart as possible in *F*_1_.

The intuition behind Heuristic 2 is that if *u* and *v* are far apart in *F*_1_, then either *u* and *v* fall into two different connected components of *F*_1_ so that we do not have to try Case 3 in the Extending Whidden *et al.*’s Algorithm section, or *u* and *v* fall into the same connected component of *F*_1_ and we can detach a lot of subtrees from *F*_1_ in Case 3.

Finally, to enumerate all MAAFs of *T*_1_ and *T*_2_, we initialize k = 0 and then proceed as follows. 

1. Simulate the sped-up algorithm for GAF on input (_*T*1_,_*T*2_,*k*,_*T*1_). During the simulation, whenever an AF *F* of *T*_1_ and *T*_2_ is enumerated, perform one of the following steps depending on whether *F* is acyclic or not: 

(a) If *F* is acyclic, output it.

(b) If *F* is cyclic, then output all AAFs *F’* of *T*_1_ and *T*_2_ such that *F’* can be obtained from *F* by performing *k*−|*F*| detaching operations on *F*.

2. If at least one AAF of *T*_1_ and *T*_2_ was outputted in Step 11a or 11b, then stop; otherwise, increase *k* by 1 and go to Step 1.

Note that Step 11b is nontrivial. As described in the supplementary material of [19], Lemma 2 is very helpful for this purpose. More specifically, we first find a cycle *C* in _*G*^*F″*^_ in *O*(|^*F″*^^|2^) time. By Lemma 2, in order to make *F’* acyclic, we have to choose one node *r* of *C* and modify *F’* by detaching the subtree of *F’* rooted at an (arbitrary) child of *r*. Note that since *r* is a root of *F’*, detaching the subtree of *F’* rooted at a child of *r* is achieved by simply deleting *r* from *F’* and is hence independent of the choice of the child. Moreover, if the parent *r’* of the dummy leaf in *F’* is a node of *C*, then by Lemma 3, we can exclude *r’* from consideration when choosing *r*. So, we have at most |^*F″*^|≤*k*−1 ways to break *C*. After modifying *F’* in this way, we again construct *G*_*F’*_ and test if it is acyclic. If it is acyclic, then we can output *F’*; otherwise, we again find a cycle *C* in *G*_*F’*_ and use it to modify *F’* as before. We repeat modifying *F’* in this way, until either *F’* becomes acyclic, or |^*F″*^|=*k*and *G*_*F’*_ is still cyclic. Once *F’* becomes acyclic, we output it. The total time taken by Step 11b is *O*(^*k*2^^(*k*−1)*k*−|^*F″*^|^), because we make a total number of at most *O*(^(*k*−1)*k*−|^*F″*^|^) choices for breaking cycles.

Experiments show that Heuristics 1 and 2 help us speed up the algorithm substantially. However, the two heuristics may not help in the worst case. That is, we are unable to prove that the two heuristics improve the worst-case time complexity of the algorithm which is *O*(^3*d*^|*X*| + ^3*d*^^(*k*−1)*k*−*d* + 2^) (as shown in [19]), where *d* is the size of an MAF of *T*_1_ and *T*_2_. We note that *k* and *d* are usually quite close.

### The new algorithm for HNE

In this subsection, we only design an algorithm for HNE. Note that it is trivial to obtain a faster algorithm for HNC by modifying the algorithm for HNE so that it stops immediately once an MAAF is found.

Throughout this subsection, let *T*_1_ and *T*_2_ be two phylogenetic trees on the same set *X* of species. As mentioned before, we can easily use an MAAF of *T*_1_ and *T*_2_ to construct a hybridization network displaying *T*_1_ and *T*_2_[6]. So, we only explain how to enumerate all MAAFs of *T*_1_ and *T*_2_.

In the last subsection, we have explained how to speed up *HybridNet* so that it can enumerate all MAAFs of *T*_1_ and *T*_2_ within shorter time. Indeed, we can make *HybridNet* even faster. The idea is to preprocess *T*_1_ and *T*_2_ so that the given trees become smaller or the problem becomes to solve two or more smaller independent subproblems. More specifically, we perform the following two reductions on *T*_1_ and *T*_2_ until neither of them is available.

#### Subtree reduction

Suppose that *T*_1_ has a non-leaf node *v*_1_ and *T*_2_ has a non-leaf node *v*_2_ such that the subtree of *T*_1_ rooted at *v*_1_ is identical to the subtree of *T*_2_ rooted at *v*_2_. Then, we modify *T*_1_ (respectively, *T*_2_) by merging the subtree of *T*_1_ (respectively, *T*_2_) rooted at *v*_1_ (respectively, *v*_2_) into a single leaf whose label set is the union of the label sets of the merged leaves. It is known [2] that this reduction preserves the MAAFs of *T*_1_ and *T*_2_.

#### Cluster reduction

Suppose that subtree reductions on *T*_1_ and *T*_2_ are not available but *T*_1_ has a non-leaf node *T*_1_ and *T*_2_ has a non-leaf node *T*_2_ such that the subtree of *T*_1_ rooted at *T*_1_ has the same leaf set as the subtree of *T*_2_ rooted at *T*_2_. Then, we split *T*_1_ (respectively, *T*_2_) into two trees *T’*_1_ and *T”*_1_ (respectively, *T’*_2_ and *T”*_2_) as follows. *T’*_1_ (respectively, *T’*_2_) is simply the subtree of *T*_1_ (respectively, *T*_2_) rooted at *T*_1_ (respectively, *T*_2_), while *T”*_1_ (respectively, *T”*_2_) is obtained by merging the subtree *T*_1_ (respectively, *T*_2_) rooted at *T*_1_ (respectively, *T*_2_) into a single leaf whose label set is the union of the label sets of the merged leaves. It is known [20] that the set of MAAFs of *T*_1_ and *T*_2_ is the Cartesian product of the set of MAAFs of *T’*_1_ and *T’*_2_ and the set of MAAFs of *T”*_1_ and *T”*_2_.

After the preprocessing stage, if no cluster reduction has been performed in the preprocessing stage, then we run the sped-up *HybridNet* (as described in the last subsection) on *T*_1_ and *T*_2_; otherwise, we have obtained two or more subproblems. Suppose that we have *h* subproblems and the *i*th subproblem (1≤*i*≤*h*) is to enumerate all MAAFs of two trees _*T*1,*i*_and _*T*2,*i*_. Then, for each 1≤*i*≤*h*, we run the sped-up *HybridNet* to enumerate the set _*i*_of MAAFs of _*T*1,*i*_ and _*T*2,*i*_. Finally, we output the Cartesan product _1_×⋯×_*h*_.

## Competing interests

The authors declare that they have no competing interests.

## Author’s contributions

ZZC was in charge of algorithm design, algorithm implementation, design of experiments, and manuscript preparation. LW participated in algorithm design and manuscript editing. SY was involved in algorithm implementation and testing. All authors read and approved the final manuscript.
